# Computational modeling for enhanced reliability in space missions: An integrated FAHP-COPRAS approach to supplier selection

**DOI:** 10.1371/journal.pone.0333310

**Published:** 2025-10-15

**Authors:** Bo-Yu Lin, Hsiaoping Yeh

**Affiliations:** 1 College of Management, National Kaohsiung University of Science and Technology, Kaohsiung, Taiwan; 2 SUMEEKO Industries Co., Ltd., Kaohsiung, Taiwan; Van Lang University: Truong Dai hoc Van Lang, VIET NAM

## Abstract

Liquid Crystal Displays (LCDs) are indispensable in space science, playing critical roles in spacecraft instrumentation, data visualization, and control systems. Selecting reliable suppliers for LCD equipment is vital to ensuring optimal performance and durability in the challenging conditions of outer space. This paper presents a comprehensive decision-making framework using fuzzy multi-criteria decision-making (MCDM) methodologies tailored for aerospace applications. The framework begins with the Fuzzy Analytic Hierarchy Process (FAHP) to determine criteria weights such as technical specifications, environmental resistance, quality and reliability, cost and delivery performance, compliance, and certifications. These criteria are crucial for meeting the stringent requirements of space missions and reflect objective metrics and expert opinions. Subsequently, the Complex Proportional Assessment of Alternatives (COPRAS) is applied to rank potential suppliers based on their performance against the weighted criteria. COPRAS allows for a comparative analysis considering positive and negative preferences, ensuring suppliers meet technical specifications and align with strategic mission objectives and constraints. Integrating FAHP and COPRAS enhances supplier selection processes’ transparency, consistency, and objectivity in aerospace procurement. This approach mitigates the risks associated with supplier variability, ensuring continuity in operations critical to space exploration and scientific advancements. The study contributes to advancing decision support systems in aerospace procurement, emphasizing rigorous supplier evaluation methodologies to enhance mission success and reliability in space science applications.

## 1. Introduction

Liquid crystal display (LCD) technology in space science faces many unique challenges, such as high radiation and large temperature fluctuations, requiring special designs to resist and adapt to harsh environments. LCDs must be equipped with anti-radiation protection to prevent damage to pixels and must be able to withstand extreme temperatures without freezing or becoming too liquid. Mechanical durability is also important; the display must withstand strong vibrations from launch and continuous operation in space. In addition, these displays need high brightness and resolution to ensure astronauts can read information easily under all lighting conditions. Besides, the power supply in space is very limited, so LCDs must be designed for low power consumption, contributing to optimizing the energy efficiency of spacecraft or space stations. These innovations help displays operate efficiently and play an important role in maintaining the integrity and reliability of displayed information, which are essential to the safety and success of space missions [1,2].

**Fig 1:**
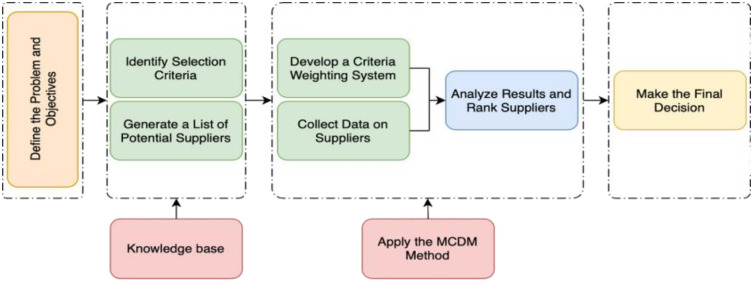
The general process of the MCDM model.

In modern space technology applications, choosing an LCD supplier becomes extremely important due to the high requirements for reliability and performance in harsh space environments. Key criteria for evaluating suppliers include resistance to radiation and extreme temperatures, product quality, technological innovation, technical support, and compliance with industry standards. The multiple criteria decision method (MCDM) is a useful tool to handle this problem, allowing for a comprehensive evaluation of suppliers based on various quantitative and qualitative criteria [3]. Fig 1 illustrates the general process of multi-criteria decision-making (MCDM) in supplier selection. The process begins with defining the problem and objectives, which sets the foundation for subsequent analysis. Next, decision-makers identify relevant selection criteria and generate a list of potential suppliers, forming the knowledge base. The following step is to develop a weighting system for the criteria and collect supplier data, which allows the application of the chosen MCDM method. Afterward, results are analyzed, and suppliers are ranked according to their performance. Finally, the process concludes with making the final decision on the most suitable supplier. This structured approach ensures transparency, consistency, and systematic evaluation in procurement contexts.

Selecting the right LCD screen supplier is a critical task to ensure both efficiency and safety in crewed space exploration missions. However, this decision is highly complex as it involves multiple interrelated factors. To address this challenge, the present study proposes a hybrid fuzzy MCDM framework that combines the Fuzzy Analytic Hierarchy Process (FAHP) and the Complex Proportional Assessment (COPRAS) method. Specifically, FAHP is applied to determine the weights of evaluation criteria, while COPRAS is used to rank and select suppliers. The main objective is to develop and characterize this integrated model, thereby enhancing the effectiveness and reliability of supplier selection in aerospace procurement.

The contribution of this study is clear in both theory and practice. From the academic side, it develops a hybrid FAHP – COPRAS model that makes supplier evaluation more transparent, consistent, and reliable, and can be applied not only in aerospace but also in other high-tech fields. From the industrial side, it gives decision-makers a practical tool to reduce risks, choose the right suppliers, and ensure smooth operations. This is especially important for LCD systems in spacecraft, which must work well under extreme conditions. Overall, the study offers a useful and adaptable method for supplier selection in critical, technology-intensive industries.

The remainder of this paper is organized into five sections. Section 2 reviews the related literature on fuzzy MCDM and supplier selection in high-tech industries. Section 3 explains the research methodology and the proposed FAHP – COPRAS framework. Section 4 presents the case study on aerospace procurement and discusses the evaluation results. Finally, Section 5 concludes the paper by summarizing key contributions, practical implications, and directions for future research.

## 2. Literature review

In recent years, the application of Fuzzy Multiple-Criteria Decision-Making (MCDM) methods in technology supplier selection has gained significant attention. Various studies have explored the effectiveness of fuzzy MCDM techniques in enhancing the supplier evaluation and selection process. Petrović et al. [4] compared three fuzzy MCDM methods for solving the supplier selection problem, highlighting the importance of such methods in decision-making processes. Sharma and Tripathy [5] introduced an integrated approach combining Quality Function Deployment (QFD) and fuzzy Technique for Order Preference by Similarity to Ideal Solution (TOPSIS) for supplier evaluation and selection, showcasing the potential of fuzzy MCDM in improving decision outcomes. Furthermore, Zhao et al. [6] applied an extended VIKOR method using intuitionistic fuzzy sets for supplier selection, emphasizing the role of fuzzy MCDM in handling complex decision scenarios. Chang et al. [7] developed a fuzzy optimization model for decision-making in supply chain management, demonstrating the integration of green criteria into fuzzy MCDM models for sustainable supplier selection. Yücesan et al. [8] proposed an integrated fuzzy MCDM method based on various decision-making techniques for evaluating green suppliers, underscoring the versatility of fuzzy MCDM in addressing diverse supplier selection criteria.

Moreover, Mohamed and Mohamed [9] introduced a fuzzy MCDM method for selecting green suppliers, providing insights into the practical application of such methods in real-world scenarios. Nguyen et al. [10] presented a spherical fuzzy MCDM model for wind turbine supplier selection, highlighting the adaptability of fuzzy MCDM in uncertain environments. Kurniawan et al. [11] discussed the extensive use of fuzzy set theory combined with MCDM methods for handling uncertainty in supplier selection decisions, emphasizing the robustness of fuzzy MCDM approaches.

Several relevant studies can be considered to further explore the application of Fuzzy MCDM methods in the context of technology supplier selection for Liquid-Crystal Display (LCD) products. Petrović et al. [4] proposed an integrated fuzzy-AHP and fuzzy multi-objective linear programming approach for supplier selection, incorporating criteria such as greenhouse gas emissions, costs, quality, lead time, and demand. This study highlights the comprehensive nature of fuzzy MCDM methods in evaluating and ranking suppliers based on multiple criteria. Moreover, Palanisamy and Zubar [12] applied Fuzzy AHP for supplier selection, specifically focusing on TFT-LCD manufacturers. This research showcases the practical implementation of fuzzy MCDM techniques in selecting suppliers for specific technology components like LCDs.

Additionally, Wang et al. [13] presented a fuzzy MCDM model using a hybrid approach of fuzzy AHP and data envelopment analysis for supplier evaluation and selection in a wind power plant project, demonstrating the versatility of fuzzy MCDM methods across different industries and applications. Furthermore, Gegovska et al. [14] explored green supplier selection using fuzzy MCDM methods and artificial neural networks, emphasizing the importance of integrating advanced technologies with fuzzy MCDM approaches for sustainable supplier selection. This study provides insights into how fuzzy MCDM techniques can be combined with other computational tools to enhance supplier selection, particularly in environmentally conscious industries like LCD manufacturing. For instance, Secundo et al. [15] demonstrated the practical application of a hybrid fuzzy extended AHP approach in an aerospace company for selecting service suppliers, specifically for a Test Data Management System (TDMS). This study showcases the successful implementation of fuzzy MCDM methods in a specialized industry like aerospace, underscoring the significance of tailored supplier selection processes for space science projects.

Liang and Chong [16] also emphasized the importance of developing new supplier selection methods that integrate various MCDM techniques modified with fuzzy set theory to address the inherent uncertainty and subjectivity in decision-making processes involving multiple stakeholders. This approach could be particularly advantageous in space science, where supplier selection criteria may vary significantly based on project requirements and technological specifications. Furthermore, Yücesan et al. [17] proposed an integrated Best-Worst and Interval Type-2 TOPSIS methodology for green supplier selection, emphasizing the importance of incorporating sustainability criteria in supplier evaluation. This aligns with the increasing focus on sustainability and environmental considerations in space science projects, where green practices and ethical sourcing are crucial factors in supplier selection decisions. Some previous papers include criteria as shown in Table 1:

**Table 1:** Summary of literature review.

Reference Number	Authors	Criteria	Methods
[5]	Sharma and Tripathy	Supplier traits	F-TOPSIS, Quality Function Deployment (QFD)
[8]	Zhao et al.	Imprecise criteria weights	Extended VIKOR, Intuitionistic Fuzzy Sets, Combination Weights
[13]	Wang et al.	Fuzzy environmental conditions	Fuzzy MCDM
[14]	Gegovska et al.	Environmental performance	Fuzzy AHP, Fuzzy TOPSIS, Fuzzy ELECTRE, ANN
[18]	Mohammed et al.	Economic, Environmental, Social	Hybrid MCDM-FMOO
[19]	Petrović et al.	Various criteria for supplier selection	Fuzzy TOPSIS, Fuzzy WASPAS, Fuzzy ARAS
[20]	Chen et al.	Economic, Environmental	Fuzzy MCDM
[21]	Nguyen et al.	Wastewater treatment, Solid waste generation, CSR, etc.	FAHP, VIKOR
[22]	Kannan et al.	Green criteria	Fuzzy MCDM
[23]	Peng et al.	Economic, Environmental, Social	Picture Fuzzy Exponential Entropy, VIKOR
[24]	Rao and Patel	Sustainable and Green criteria	Fuzzy R-method
[25]	Boltürk	Various criteria for manufacturing firm suppliers	Pythagorean fuzzy CODAS
[26]	Yucesan et al.	Environmental criteria for green supplier selection	Best-Worst Method (BWM), Interval type-2 fuzzy TOPSIS
[27]	Deniz	Various criteria to mitigate cognitive biases	Fuzzy MOORA, FMEA
[28]	Göçer	Sustainable supplier evaluation	AHP, TOPSIS, Pythagorean Fuzzy Sets (PFSs)
[29]	Gündüz et al.	Quality, Delivery time, Cost, Technology, etc.	Fuzzy TOPSIS
[30]	Uehara and Matsumaru	Various criteria for LCD space science and green supplier selection	Three-factor Fuzzy Information Channel Model
[31]	Zheng	Criteria for International Supplier Selection	ANP, VIKOR, Hesitant Fuzzy Environment
[29]	Zhang et al.	Various criteria for manufacturing services	Interval-Valued Intuitionistic Fuzzy Weighted Arithmetic (IIFWA)
[30]	Azadnia et al.	Various criteria for supplier categorization	Fuzzy C-Means (FCM) Clustering

In the realm of green supplier selection, the application of Fuzzy MCDM methods is pivotal for evaluating and choosing environmentally sustainable suppliers. Various studies have delved into fuzzy MCDM techniques in green supplier selection, emphasizing the importance of integrating sustainability criteria into supplier evaluation processes. Wang et al. [32] developed a Fuzzy MCDM model for sustainable supplier evaluation and selection in the garment industry, focusing on Triple Bottom Line approaches. This study stresses the significance of considering economic, environmental, and social criteria when assessing and ranking green suppliers, aligning with sustainability principles in supplier selection processes. Additionally, Guo et al. [33] utilized a Fuzzy Multi-Criteria Decision-Making approach for green supplier evaluation and selection in apparel manufacturing. The study reviewed green supplier selection literature and identified commonly used AHP, ANP, and TOPSIS, showcasing the various methodologies available for evaluating green suppliers. Moreover, Wang et al. [34] employed fuzzy MCDM approaches, including Fuzzy TOPSIS, Fuzzy VIKOR, and Fuzzy Grey Relational Analysis, for green supplier evaluation and selection in the agri-food industry. This research illustrates the adaptability of fuzzy MCDM methods in managing complex decision-making scenarios and integrating multiple criteria for sustainable supplier selection.

In summary, while previous studies have applied FAHP or COPRAS independently to supplier selection and other decision-making contexts, few have explored their integration in a hybrid fuzzy framework specifically tailored for high-tech and aerospace procurement. The novelty of this study lies in combining FAHP’s ability to capture expert uncertainty in weighting criteria with COPRAS’s strength in delivering transparent and robust rankings, thus overcoming the limitations of single-method approaches. By situating this hybrid model in the context of LCD supplier evaluation for space missions, the research extends the application of fuzzy MCDM methods to a domain where safety, reliability, and performance under extreme conditions are paramount. This dual emphasis on methodological integration and domain-specific application underlines the scholarly contribution and distinguishes this work from prior studies.

## 3. Methodology

This study proposes a Fuzzy Multi-Criteria Decision-Making (F-MCDM) model for selecting the most suitable sustainable supplier in the field of space sciences, employing an integrated approach based on the Fuzzy Analytic Hierarchy Process (FAHP) and the Complex Proportional Assessment of Alternatives (COPRAS) methodologies. The research is structured into three key phases:

**Phase 1**: A comprehensive set of evaluation criteria and sub-criteria influencing the selection of LCD suppliers is identified through expert consultations and an extensive review of relevant literature.

**Phase 2**: The relative importance (weights) of the identified criteria is determined using the FAHP method, capturing expert judgments under uncertainty with the support of fuzzy logic.

**Phase 3**: The COPRAS technique is applied to rank the shortlisted suppliers based on their performance against the weighted criteria. The resulting ranking serves as a decision-support tool, guiding procurement professionals in making informed and sustainable supplier choices for space applications.

### 3.1. Fuzzy theory

Number The Triangular Fuzzy Number (TFN) can be represented by the symbol (k, h, g), where the parameters k, h, and g (k ≤ h ≤ g) specify the lowest, maximum, and most likely values inside TFN.


$$\mu \left(\frac{x}{\tilde{M}}\right)=\left\{ \begin{array}{c} \text{0},\\ \frac{x-k}{h-k}\\ \frac{g-x}{g-h}\\ \text{0}, \end{array}\begin{array}{c} x<h,\\ k\leq x\leq h,\\ h\leq x\leq g,\\ x>g, \end{array}\right.$$ (1)

A fuzzy number is provided as:


$$\tilde{M}={(M}^{o(y)},M^{i(y)})$$

$$\tilde{M}=\left[k+(h-k)y,g+(h-g)y\right],y\in [\text{0},\text{1}]$$ (2)

With o(yandi(y) denoting, respectively, the left and right sides of a fuzzy value. The following illustrates how two positive TFNs, $(k_{\text{1}},h_{\text{1}},g_{\text{1}}\{a\}nd\left(k_{\text{2}},h_{\text{2}},g_{\text{2}}\right)$, are involved in basic calculations.


$$\left(k_{\text{1}},h_{\text{1}},g_{\text{1}}\right)+\left(k_{\text{2}},h_{\text{2}},g_{\text{2}}\right)=\left(k_{\text{1}}+k_{\text{2}},h_{\text{1}}+h_{\text{2}},g_{\text{1}}+g_{\text{2}}\right)$$

$$\left(k_{\text{1}},h_{\text{1}},g_{\text{1}}\right)-\left(k_{\text{2}},h_{\text{2}},g_{\text{2}}\right)=\left(k_{\text{1}}-k_{\text{2}},h_{\text{1}}-h_{\text{2}},g_{\text{1}}-g_{\text{2}}\right)$$ (3)

$$\left(k_{\text{1}},h_{\text{1}},g_{\text{1}}\right)\times \left(k_{\text{2}},h_{\text{2}},g_{\text{2}}\right)=\left(k_{\text{1}}\times k_{\text{2}},h_{\text{1}}\times h_{\text{2}},g_{\text{1}}\times g_{\text{2}}\right)$$

$$\frac{\left(k_{\text{1}},h_{\text{1}},g_{\text{1}}\right)}{\left(k_{\text{2}},h_{\text{2}},g_{\text{2}}\right)}=\left(k_{\text{1}}/k_{\text{2}},h_{\text{1}}/h_{\text{2}},g_{\text{1}}/g_{\text{2}}\right)$$

### 3.2. Display style

An expansion of AHP called the Fuzzy Analytical Hierarchy Process (FAHP) uses fuzzy set theory to determine its limitations when applied to uncertain decision-making situations. Let $K=\left\{k_{\text{1}},k_{\text{2}},\ldots .k_{n}\right\}$ be the last appropriate set, and $X=\left\{x_{\text{1}},x_{\text{2}},\ldots .x_{n}\right\}$ be the set of objects. The extent analysis method proposed by Chang [37] calculates the extent analysis of the final solution for each value taken. Consequently, retrieving the l extent analysis values for every object is possible. These numbers are represented as:


$$L_{k_{i}}^{\text{1}},L_{k_{i}}^{\text{2}},{\ldots ,L}_{k_{i}}^{m},i=\text{1},\text{2},\ldots ,n.$$ (4)

where $L_{k}^{j}(j=\text{1},\text{2},\ldots .,m)$ are the TFNs.

The fuzzy synthetic extent value of the $i^{th}$ object is deﬁned as:


$$S_{i}=\sum {\mathrm{\backslash nolimits}}_{j=\text{1}}^{m}L_{k_{i}}^{j}⊗{\left[\sum {\mathrm{\backslash nolimits}}_{i=\text{1}}^{n}\sum {\mathrm{\backslash nolimits}}_{j=\text{1}}^{m}L_{k_{i}}^{j}\right]}^{-\text{1}}$$ (5)

The possibility that $L_{\text{1}}\geq L_{\text{2}}$ is defined as:


$$V\left(L_{\text{1}}\geq L_{\text{2}}\right)={sup}_{y\geq x}[min\left(\mu_{L_{\text{1}}}(x),\right),\left(\mu_{L_{\text{2}}}(y)\right)\backslash$$ (6)

Where the pair (x,y) exists with x≥y and $\mu_{L_{\text{1}}}(x)=\mu_{L_{\text{2}}}(y)$, then we have $V\left(L_{\text{1}}\geq L_{\text{2}}\right)=\text{1}$.

since $L_{\text{1}}$ and $L_{\text{2}}$ are convex fuzzy numbers, we have):


$$\left(L_{\text{1}}\geq L_{\text{2}}\right)=\text{1},ifl_{\text{1}}\geq l_{\text{2}}$$ (7)

And


$$V\left(L_{\text{2}}\geq L_{\text{1}}\right)=hgt\left(L_{\text{1}}\cap L_{\text{2}}\right)=\mu_{L_{\text{1}}}(d)$$ (8)

Where d is the ordinate of the highest intersection point D between $\mu_{L_{\text{1}}}$ and $\mu_{L_{\text{2}}}$

With $L_{\text{1}}=(o_{\text{1}},p_{\text{1}}$, $q_{\text{1}}$ and $L_{\text{2}}=(o_{\text{2}},p_{\text{2}}$,$q_{\text{2}})$, the ordinate of point D is calculated by (9):


$$V\left(L_{\text{2}}\geq L_{\text{1}}\right)=hgt\left(L_{\text{1}}\cap L_{\text{2}}\right)=\frac{l_{\text{1}}-q_{\text{2}}}{\left(p_{\text{2}}-q_{\text{2}}\right)-\left(p_{\text{1}}-o_{\text{1}}\right)}$$ (9)

In order to compare $L_{\text{1}}$ and $L_{\text{2}}$, we need to calculate the values of $V\left(L_{\text{1}}\geq L_{\text{2}}\right)$ and $V\left(L_{\text{2}}\geq L_{\text{1}}\right)$.

The possibility for a convex fuzzy number to be greater than k convex fuzzy numbers $L_{i}(i=\text{1},\text{2},\ldots k)$ is calculated as:


$$V\left(L\geq L_{\text{1}},L_{\text{2}},\ldots ,L_{k}\right)=V\left[\left(L\geq L_{\text{1}}\right)and\left(L\geq L_{\text{2}}\right)\right]$$ (10)

and, $(L\geq L_{k}$) = min V (L$\geq L_{i}),i=\text{1},\text{2},\ldots ,k$

Under the assumption that:


$$d^{\prime }\left(B_{i}\right)=minV(S_{i}\geq S_{k})$$ (11)

for k=1,2,…nandk#i, the weight vector is determined as:


$$W^{\prime }={\left(d^{\prime }\left(B_{\text{1}}\right),d^{\prime }\left(B_{\text{2}}\right),\ldots d^{\prime }\left(B_{n}\right)\right)}^{T}$$ (12)

Where $B_{i}$ are n elements.

These are the normalized weight vectors displayed:


$$W={\left(d\left(B_{\text{1}}\right),d\left(B_{\text{2}}\right),\ldots .,d\left(B_{n}\right)\right)}^{T}$$ (13)

With W is a nonfuzzy number.

Saaty’s matrix evaluation is utilized to check for consistency.


$$CR=\frac{CI}{RI}=\frac{\overset{―}{\lambda }-n}{(n-\text{1})\times RI}\leq \text{0}.\text{1}$$ (14)

### 3.3. COPRAS method

The suggested steps [38] are the methodology’s next steps:

Step 1: Identifying and choosing the options and contributing criteria. First, the accessible options are selected, and the attributes that influence the decision in the MCDM problem are identified.

Create the decision matrix in step two, which compares options to attributes (X).

Eq. (15) indicates that the collected data (options and characteristics) are layered in a matrix construction:


$$\text{X}=\left[\begin{array}{ccc} @ccc@{\text{x}}_{\text{11}} & {\text{x}}_{\text{12}} & \ldots .{\text{x}}_{\text{1m}}\\ {\text{x}}_{\text{21}} & {\text{x}}_{\text{22}} & \ldots .{\text{x}}_{\text{2m}}\\ : & : & :\\ {\text{x}}_{\text{n1}} & {\text{x}}_{\text{n2}} & {\text{x}}_{\text{nm}} \end{array}\right]$$ (15)

where n= number of options; m = number of attributes

Step 2: Normalization of decision matrix ($\overset{―}{\text{X}})$

Equation (16) displays the decision’s normalization.


$$\overset{―}{\text{X}}=\left[\begin{array}{ccc} @ccc@{\overset{―}{\text{x}}}_{\text{11}} & {\overset{―}{\text{x}}}_{\text{12}} & \ldots .{\overset{―}{\text{x}}}_{\text{1m}}\\ {\overset{―}{\text{x}}}_{\text{21}} & {\overset{―}{\text{x}}}_{\text{22}} & \ldots .{\overset{―}{\text{x}}}_{\text{2m}}\\ : & : & :\\ {\overset{―}{\text{x}}}_{\text{n1}} & {\overset{―}{\text{x}}}_{\text{n2}} & {\overset{―}{\text{x}}}_{\text{nm}} \end{array}\right]$$ (16)

Where ${\overset{―}{\text{x}}}_{\text{ij}}$=$\frac{{\text{x}}_{\text{ij}}}{\sum_{\text{i}=\text{1}}^{\text{n}}{\text{x}}_{\text{ij}}}$; I = 1, 2, …..n; and j = 1, 2, … m

Step 3: Calculation of the weighting of the attributes (${\text{W}}_{\text{j}}$)

The attributes weightings are calculated by using FAHP calculations.

Step 4: Calculation of the overall normalized matrix $\left(\hat{\text{X}}\right)$

To obtain the overall normalized matrix, the computed weights are multiplied by the matching attribute value of each option


$$\hat{\text{X}}=\left[\begin{array}{ccc} @ccc@{\hat{\text{x}}}_{\text{11}} & {\hat{\text{x}}}_{\text{12}} & \ldots .{\hat{\text{x}}}_{\text{1m}}\\ {\hat{\text{x}}}_{\text{21}} & {\hat{\text{x}}}_{\text{22}} & \ldots .{\hat{\text{x}}}_{\text{2m}}\\ : & : & :\\ {\hat{\text{x}}}_{\text{n1}} & {\hat{\text{x}}}_{\text{n2}} & {\hat{\text{x}}}_{\text{nm}} \end{array}\right]$$ (17)

Where ${\hat{\text{X}}}_{\text{ij}}={\overset{―}{\text{x}}}_{\text{ij}}*{\text{W}}_{\text{j}}$

Step 5: Determination of maximizing index (${\text{P}}_{\text{j}}$) and minimizing index (${\text{R}}_{\text{j}}$)

The values of the maximizing index (${\text{P}}_{\text{j}}$) and minimizing index (${\text{R}}_{\text{j}}$) are established based on the qualitative nature of the attribute. If attribute (${\text{P}}_{\text{j}}$) is a maximizing index, it is found. We’ll compute (${\text{R}}_{\text{j}}$)to minimize the index.


$${\text{P}}_{\text{j}}=\sum {\mathrm{\backslash nolimits}}_{\text{i}=\text{1}}^{\text{k}}{\hat{\text{x}}}_{\text{ij}}$$ (18)

$${\text{R}}_{\text{j}}=\sum {\mathrm{\backslash nolimits}}_{\text{i}=\text{k}+\text{1}}^{\text{m}}{\hat{\text{x}}}_{\text{ij}}$$ (19)

where k = number of attributes which is to be maximized

Step 7: Calculation of relative weights of each option (${\text{Q}}_{\text{j}}$)

Finally, all the attributes’ overall relative weighting will be established.


$$Q_{j}=P_{j}+\frac{\sum_{j=\text{1}}^{n}R_{j}}{R_{j}\sum_{j=\text{1}}^{n}\frac{\text{1}}{R_{j}}}$$ (20)

The greatest option is the one that has the highest relative weights among the alternatives.

## 4. Case study

Liquid Crystal Display plays an important role in space exploration projects thanks to its ability to display data and images from devices and sensors, helping astronauts and scientists monitor the status of equipment and the surrounding environment and perform scientific experiments. It provides an intuitive human-machine interface for control systems, helping astronauts and engineers easily interact with complex equipment. LCDs also help monitor astronauts’ health by displaying important medical indicators. In particular, LCDs can adjust brightness and contrast to improve visibility in the harsh and constantly changing space environment. Furthermore, LCDs consume less power than other display technologies, helping to save energy, which is very important in space missions. These features make LCDs essential, supporting operations and providing necessary information for space exploration projects.

Selecting equipment providers for space exploration projects is crucial since this equipment needs to function reliably in hostile environments, including intense radiation and extreme temperatures. High technical standards, a willingness to innovate in technology, and comprehensive technical assistance are requirements for suppliers. They must simultaneously uphold safety and environmental requirements. Not only that, but the cost of acquisition, upkeep, and replacement, as well as the economic worth of the solutions they offer, must be considered. In this study, the authors proposed a Multi-criteria Decision-Making Model including the Fuzzy Analytic Hierarchy Process (FAHP) approach and the Complex Proportional Assessment of Alternatives (COPRAS) model for Liquid Crystal Display supplier evaluation and selection in space science projects.

Four suppliers (LCDS1, LCDS2, LCDS3, and LCDS4) are considered based on their experience and advanced technology to produce high-quality LCDs that meet the special requirements of space projects, including the ability to operate in harsh environments and efficient energy consumption. In the first stage, the author applied the FAHP model to define the weight of the criteria. A list of criteria is shown in Table 2.

**Table 2:** List of criteria.

Criteria	Subcriteria	Symbol
LCD1: Technical Specifications	Resolution, Brightness, and Contrast	LCD1.1
	Durability, Viewing Angle	LCD1.2
	Response Time	LCD1.3
LCD2: Environmental Resistance	Temperature Range	LCD2.1
	Radiation Resistance	LCD2.2
	Pressure Resistance	LCD2.3
	Electromagnetic Interference (EMI) Shielding	LCD2.4
LCD3: Quality and Reliability	Manufacturing Quality	LCD3.1
	Reliability	LCD3.2
	Compliance with Standards	LCD3.3
LCD4: Cost and Delivery Performance	Initial Cost	LCD4.1
	Lead Time	LCD4.2
	Flexibility	LCD4.3
LCD5: Compliance and Certifications	Regulatory Compliance	LCD5.1
	Certifications	LCD5.2

The FAHP model provides a flexible and comprehensive approach to calculating weights in situations with many uncertain factors, helping decision-makers make more accurate choices. The weights of the fifteen criteria are shown in Table 3:

**Table 3:** List of criteria.

Criteria	Symbol	Weight
Resolution, Brightness, and Contrast	LCD1.1	0,055
Durability, Viewing Angle	LCD1.2	0,054
Response Time	LCD1.3	0,074
Temperature Range	LCD2.1	0,088
Radiation Resistance	LCD2.2	0,105
Pressure Resistance	LCD2.3	0,064
Electromagnetic Interference (EMI) Shielding	LCD2.4	0,051
Manufacturing Quality	LCD3.1	0,083
Reliability	LCD3.2	0,087
Compliance with Standards	LCD3.3	0,050
Initial Cost	LCD4.1	0,050
Lead Time	LCD4.2	0,099
Flexibility	LCD4.3	0,046
Regulatory Compliance	LCD5.1	0,038
Certifications	LCD5.2	0,058

To illustrate the practical application of the proposed FAHP – COPRAS framework, four potential LCD suppliers were evaluated based on their suitability for aerospace missions. These suppliers were pre-selected through an initial screening process, ensuring compliance with fundamental technical and regulatory requirements. Table 4 presents an overview of the key characteristics of each supplier, highlighting differences in experience, certifications and technological strengths.

**Table 4:** Summary of Key Characteristics of the Evaluated LCD Suppliers for Aerospace Applications.

Supplier	Experience	Certifications	Key Features
LCDS1	>15 years in LCDs for aerospace	ISO 9001, AS9100	Highly customized for radiation resistance and thermal stability; superior quality and reliability
LCDS2	10 years supplying LCDs for harsh conditions	MIL-STD-810	Cost-effective, fast delivery, designed for low-pressure and high-altitude conditions
LCDS3	Research collaborations with space labs	ISO 14001	Energy-efficient, high-contrast LCDs with anti-reflective and anti-vibration coatings
LCDS4	Recently expanded from consumer electronics	ISO 9001 (for commercial use)	Modular integration, low cost, adapted commercial LCDs for aerospace use

After determining the relative importance of the evaluation criteria using the Fuzzy Analytic Hierarchy Process (FAHP), the Complex Proportional Assessment (COPRAS) method was employed to rank the shortlisted LCD suppliers. COPRAS is particularly suitable for complex decision-making scenarios in aerospace procurement, as it simultaneously considers both beneficial and non-beneficial criteria, ensuring a balanced evaluation. Unlike traditional MCDM approaches that may overlook negative aspects, COPRAS explicitly integrates disadvantageous attributes such as high cost or poor environmental resistance—into the decision-making process, enhancing the objectivity of supplier comparisons. This feature is especially critical in space science applications, where component failure can lead to mission-critical consequences. By quantifying and aggregating the weighted performance of each supplier across all criteria, COPRAS enables a transparent, systematic, and reproducible ranking process that aligns technical excellence with operational feasibility. The results of the COPRAS model are shown in Table 5.

**Table 5:** Results of the COPRAS model.

Alternatives	S+	S-	1/S-	Q	U	Ranking
LCDS1	0.2716	0.0141	71.1111	0.2825	100.00	1
LCDS2	0.2151	0.0109	91.4286	0.2292	81.13	3
LCDS3	0.2379	0.0141	71.1111	0.2488	88.06	4
LCDS4	0.2454	0.0109	91.4286	0.2595	91.84	2

Based on the COPRAS model in Table 5, four suppliers were ranked, and the supplier with the symbol LCDS1 was found to be the most appropriate. The COPRAS model considers several factors and rates each supplier according to how well they meet these requirements. Although LCDS1 performed the best overall in this instance, the model acknowledges that other providers might also be a good choice in some circumstances.

**Fig 2:**
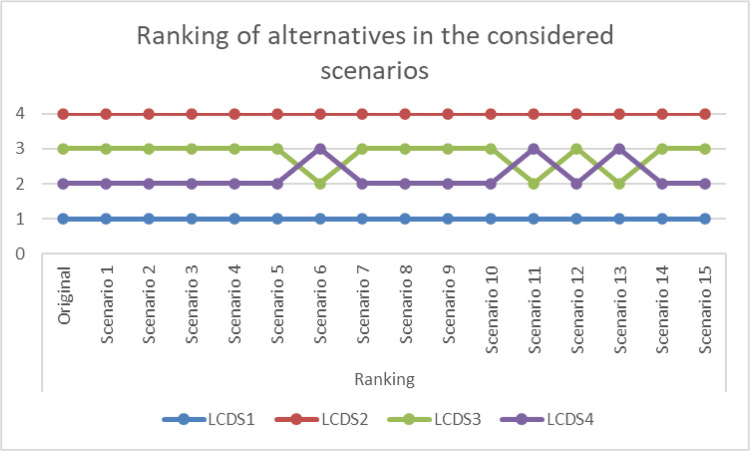
Alternatives rankings in all scenarios.

A sensitivity analysis is performed to evaluate the outcome of the proposed method. One approach to robust testing and sensitivity analysis is to calculate the final ranking of alternatives when the weight of a specific criterion is altered. In this case, the weight of each is reduced to 0, and the impact on the final ranking is examined using the procedure outlined by Alinezhad and Amini [31]. The performance scores of the alternatives are shown in Table 6 and their rankings are shown in Fig 2.

**Table 6:** Alternative performance scores in all scenarios.

Alternatives	Performance Score															
	Original	Scenario 1	Scenario 2	Scenario 3	Scenario 4	Scenario 5	Scenario 6	Scenario 7	Scenario 8	Scenario 9	Scenario 10	Scenario 11	Scenario 12	Scenario 13	Scenario 14	Scenario 15
LCDS1	100.00	100.00	100.00	100.00	100.00	100.00	100.00	100.00	100.00	100.00	100.00	100.00	100.00	100.00	100.00	100.00
LCDS2	81.13	79.70	80.85	81.49	82.77	80.74	89.70	82.11	80.51	80.47	81.36	86.24	82.93	88.52	88.40	88.14
LCDS3	88.06	86.50	87.17	88.44	89.64	84.45	95.58	89.85	89.42	87.39	87.89	92.36	89.76	93.95	92.75	94.09
LCDS4	91.84	88.33	92.63	93.90	93.24	90.89	92.97	92.60	90.99	93.03	91.30	92.11	92.06	93.16	93.23	95.05

Fig 2 show that alternative LCDS1 is the optimal choice in every case. This indicates that, despite some changes in the ranking of other alternatives, LCDS consistently performs well across all scenarios, and its ranking is robust against changing criteria weights.

The sensitivity analysis provides important practical insights for decision-makers. The fact that LCDS1 remains the top-ranked supplier across all scenarios implies that its selection is stable and resilient, even when there are uncertainties or shifts in the relative importance of evaluation criteria. In practice, this means that managers in aerospace procurement can make supplier decisions with greater confidence, knowing that the outcome is not overly sensitive to subjective judgments or minor changes in weight assignments. Moreover, the observed variations in the rankings of other suppliers highlight where potential risks or trade-offs may arise, allowing procurement teams to identify backup options and prepare contingency strategies. This practical robustness is particularly valuable in high-stakes contexts such as space missions, where reliability and consistency are critical.

## 5. Conclusion

This study has proposed and validated a novel decision-making framework integrating Fuzzy Analytic Hierarchy Process (FAHP) and Complex Proportional Assessment of Alternatives (COPRAS) to support supplier selection in aerospace applications, particularly for Liquid Crystal Display (LCD) systems. The framework effectively addresses the complexity and uncertainty inherent in evaluating technical suppliers for space missions, where performance, reliability, and environmental resistance are paramount.

The findings of this study have several important implications for managers and engineers working in aerospace procurement. First, the proposed FAHP – COPRAS model enables procurement teams to evaluate LCD suppliers more systematically by balancing technical specifications, quality assurance, compliance, and environmental performance. This reduces reliance on subjective judgment and provides a transparent framework for supplier selection. Second, the robustness of LCDS1’s ranking across sensitivity scenarios assures decision-makers that the selected supplier can perform reliably even when there are uncertainties or changes in evaluation priorities. For engineers, this means greater confidence in the operational stability of critical LCD systems, which must function effectively under extreme conditions of temperature, vibration, and radiation in space. For managers, the framework highlights not only the optimal supplier but also alternative options, allowing the development of contingency plans in case of supply chain disruptions. By demonstrating both reliability and flexibility, the proposed model offers a practical decision-support tool that directly contributes to risk reduction, operational continuity, and the overall success of space exploration missions.

Future studies could extend this work by integrating other fuzzy MCDM techniques such as fuzzy TOPSIS, DEMATEL, or VIKOR to better capture interdependencies among criteria and provide alternative decision perspectives. Another promising direction is adapting the framework to dynamic contexts, where supplier evaluation criteria may change with evolving space mission requirements. Moreover, extending the framework to consider dynamic criteria under changing space mission requirements or integrating real-time data from supplier performance monitoring systems could add significant value to long-term aerospace procurement strategies.
